# Hyperoxia-Induced Protein Alterations in Renal Rat Tissue: A Quantitative Proteomic Approach to Identify Hyperoxia-Induced Effects in Cellular Signaling Pathways

**DOI:** 10.1155/2015/964263

**Published:** 2015-05-27

**Authors:** Jochen Hinkelbein, Lennert Böhm, Oliver Spelten, David Sander, Stefan Soltész, Stefan Braunecker

**Affiliations:** ^1^Department for Anaesthesiology and Intensive Care Medicine, University Hospital of Cologne, 50937 Cologne, Germany; ^2^Department for Anaesthesia, Dormagen Hospital, 41540 Dormagen, Germany

## Abstract

*Introduction*. In renal tissue as well as in other organs, supranormal oxygen pressure may lead to deleterious consequences on a cellular level. Additionally, hyperoxia-induced effect in cells and related free radicals may potentially contribute to renal failure. The aim of this study was to analyze time-dependent alterations of rat kidney protein expression after short-term normobaric hyperoxia using proteomics and bioinformatic approaches. *Material and Methods*. *N* = 36 Wistar rats were randomized into six different groups: three groups with normobaric hyperoxia (exposure to 100% oxygen for 3 h) and three groups with normobaric normoxia (NN; room air). After hyperoxia exposure, kidneys were removed immediately, after 3 days and after 7 days. Kidney lysates were analyzed by two-dimensional gel electrophoresis followed by peptide mass fingerprinting using tandem mass spectrometry. Statistical analysis was performed with DeCyder 2D software (*p* < 0.01). Biological functions of differential regulated proteins were studied using functional network analysis (Ingenuity Pathways Analysis and PathwayStudio). *Results*. Expression of 14 proteins was significantly altered (*p* < 0.01): eight proteins (MEP1A_RAT, RSSA_RAT, F16P1_RAT, STML2_RAT, BPNT1_RAT, LGMN_RAT, ATPA_RAT, and VDAC1_RAT) were downregulated and six proteins (MTUS1_RAT, F16P1_RAT, ACTG_RAT, ACTB_RAT, 2ABA_RAT, and RAB1A_RAT) were upregulated. Bioinformatic analyses revealed an association of regulated proteins with inflammation. *Conclusions*. Significant alterations in renal protein expression could be demonstrated for up to 7 days even after short-term hyperoxia. The identified proteins indicate an association with inflammation signaling cascades. MEP1A and VDAC1 could be promising candidates to identify hyperoxic injury in kidney cells.

## 1. Introduction

Oxygen toxicity is a well-known phenomenon that may result in several severe symptoms like seizures and convulsions, cardiovascular and gastrointestinal disorders, erythrocyte damage, and retinopathy [1, 2]. Furthermore, arterial hyperoxia may be associated with an increased mortality in critically ill patients [3]. While hyperoxia of various organs (e.g., lungs, brain, or retina) has been investigated sufficiently in recent years [4, 5], molecular effects and pathway alterations in renal tissue and kidneys due to hyperoxia are rarely studied. Whereas there is growing evidence that normobaric hyperoxia has significant effects in preterm or neonatal kidneys [6, 7], data for adults are lacking.

In general, hyperoxia (NH) leads to an overwhelming production of reactive oxygen species (ROS; •O_2_^−^) which in turn initiate an inflammatory cellular response. The degree of damage caused by hyperoxia depends on the balance between ROS production and the capacity of renal antioxidant systems [8, 9]. A linear increase of ROS concentration with oxygen tension in mitochondria has been observed [10]. •O_2_^−^ is generated by the mitochondrial electron transport chain by the transfer of an electron to molecular O_2_.

As another source of ROS, the nicotinamide adenine dinucleotide phosphate (NADPH) oxidase (NOX) family utilizes NADPH/NADH as an electron donor to catalyze reduction of molecular oxygen to •O_2_^−^ at the extracellular side of the plasma membrane [11, 12]. These hyperoxia-induced effects are mediated by complex sets of genes and their products rather than being the result of only one single gene.

The type and degree of ROS damage to kidneys and renal tissue have not been investigated in detail yet. There are some studies suggesting that high oxygen concentrations in inspired air as well as resulting hyperoxia may lead to detrimental renal effects, at least in neonatal rats [6]. However, results of previous studies on beneficial as well as detrimental effects of hyperoxia are still controversially discussed [2, 13, 14].

Although important molecules have been investigated individually, a comprehensive analysis of protein expression during and after hyperoxia exposure in the kidney has not been performed yet but would improve our knowledge of local and systemic effects. These effects may also play a role during inflammation or sepsis [15–17].

The present study used the proteomic methods of two-dimensional gel electrophoresis (2D-DIGE), peptide mass fingerprinting via matrix-assisted laser desorption/ionization time-of-flight tandem mass spectrometry (MALDI-TOF MS/MS), and bioinformatic molecular network analyses to investigate some of the complex renal protein alterations in rats after short-term hyperoxia. These data may help to understand, and possibly prevent, hyperoxic renal injury in the future. It was not intended to find specific biomarkers for hyperoxia but to find affected pathways for future biomarker research.

## 2. Material and Methods

### 2.1. Animals

Experiments were approved by the local institutional animal research review board (Regierungspräsidium Karlsruhe, Germany; AZ 35-9185.81/G-56/04) and were performed according to the regulations of the National Institutes of Health (NIH) guide for the use of laboratory animals. Thirty-six male Wistar rats (Charles River Deutschland, Sulzfeld, Germany) were kept under temperature controlled environmental conditions at 22°C on a 14 h light-cycle followed by a 10 h dark-cycle prior to the experiments and were fed a standard diet (Altromin C1000; Altromin, Lage, Germany) with free access to food and water.

### 2.2. Study Groups

**Figure 1:**
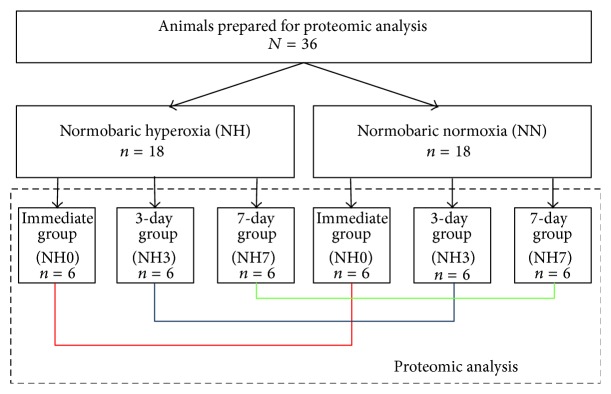
Animals analyzed and assigned to six different groups (3 groups with hyperoxia (days 0, 3, and 7) and 3 groups with normoxia (days 0, 3, and 7)). Corresponding groups for day 0, day 3, and day 7 were compared to each other. Animals in the normoxia groups served as controls.

A total of *N* = 36 rats weighing 280 ± 21 g were used in this study (Figure 1) and prepared for proteomic analysis. After weighing, 18 animals were randomly assigned to three NH groups and the remaining 18 rats were allocated to three normobaric normoxia groups (NN). Following this group formation, all animals (NH and NN) were randomly assigned to one specific subgroup: ‘‘immediate analysis” (NH0 or NN0; day 0), ‘‘analysis at day 3” (NH3 or NN3), or ‘‘analysis at day 7” (NH7 or NN7). This resulted in six different groups with six animals each (Figure 1).

### 2.3. Hyperoxia Exposure

At the beginning of the experiments, rats were placed into an air-sealed box (30 × 18 × 18 cm) with one small inlet hole and one small outlet hole. For animals receiving hyperoxia, an oxygen line was connected to the inlet providing a flow of 5 L min^−1^ of pure medical oxygen (concentration 99.5%; medicAL, Air Liquide, Duisburg, Germany).

Rats of the normoxia groups received 5 L min^−1^ room air until the end of the experiments. Oxygen concentration was measured continuously in the box for both groups. After 3 h of hyperoxia or normoxia, respectively, experiments were terminated and the animals were immediately removed from the box.

The rats of the NH3, NH7, NN3, and NN7 groups were placed back into their cages and provided with food and water ad libitum until the planned end of the experiments (3 or 7 days after the experimental start). Rats of the NH0 and NN0 groups reached the defined end-point of the experiments immediately.

At the defined end-points of the experiments, the animals of the specific group (immediately, 3 or 7 days after exposure to 3-h hyperoxia) were quickly anesthetized with Sevorane (Sevoflurane, Baxter, Unterschleissheim, Germany) in a concentration of 5% at room air and underwent both cardiac puncture for blood removal, which was used for analysis of arterial blood gases, electrolytes, hemoglobin (Rapidlab 865, Bayer Vital Diagnostics; Fernwald, Germany), blood cell count (Advia 60; Bayer Vital Diagnostics; Fernwald, Germany), albumin, and phosphate (Advia 2400; Bayer Vital Diagnostics; Fernwald, Germany).

Afterwards, the abdomen was opened carefully and the kidneys were removed for proteomic analysis as quickly as possible, frozen in isopentane prechilled to −40 to −50°C, and stored at −80°C until further analysis.

### 2.4. Sample Preparation

Each frozen kidney sample was weighed and cut into smaller pieces. The frozen tissues of the six samples from each time-point were pooled and grinded with liquid nitrogen in a mortar. The tissue powder of the pooled samples was mixed with 7 ml of lysis buffer (7 M urea, 2 M thiourea, 4% Chaps, 30 mM Tris pH 8.5, Roche Complete Protease Inhibitor Cocktail, 1.2% Pefablock SC Protease Inhibitor, 1% Sigma Protease Inhibitor Cocktail 2, 1% Sigma Protease Inhibitor Cocktail 3) and transferred into a 15-ml reaction tube. Cells were lysed and proteins dissolved using vigorous vortexing and sonication with 15 pulses of 1 second on ice. Samples were centrifuged for 10 min at 10 000 ×g to pellet debris and insoluble material. The supernatant was taken for further analysis.

The protein concentration of samples was determined using a Bradford Assay [18]. According to the protein determination results with Bradford assay, 200 *µ*g protein of each sample was diluted with labeling buffer (30 mM Tris, pH 8.5; 7 M urea; 2 M thiourea, 4% Chaps, Roche Complete Protease Inhibitor Cocktail, Pefablock SC Protease Inhibitor) to a final volume of 450 *µ*l. The samples were desalted and rebuffered with labeling buffer using Vivaspin 500 ultrafiltration devices with a cut-off of 5 kDa. After volume reduction samples were present in 60 *µ*l labeling buffer at last.

### 2.5. DIGE Labeling

From each sample 50 *µ*g protein was labeled with Cyanine Dye (Cy, CyDye) 3 (Cy3) and 50 *µ*g protein was labeled with Cy5. Additionally, samples were pooled as the internal standard sample and 50 *µ*g (15 *µ*l; 15.1%) of this mixture was used for each labeling reaction with Cy2. For each labeling reaction with Cy2, Cy3, and Cy5 50 *µ*g protein (15 *µ*l) was applied to 400 pmol CyDye. The reactions were performed on ice for 30 min and stopped by adding lysine and incubating for another 10 min. Labeling was performed according to the manufacturers protocol (FluoProbes/InterChim:* 2D DIGE Cy5/3/2 Labeling Kit*). The labeling chemistry is based on minimal labeling of lysine residues and primary amino groups of the proteins, with one lysine residue labeled per protein on average [19].

After labeling, reactions to run in the same 2D-DIGE gel were pooled. The final volume of each sample was adjusted to 120 *µ*l with lysis buffer B (7 M urea; 2 M thiourea; 4% Chaps; Roche Complete Protease Inhibitor Cocktail, Pefablock SC Protease Inhibitor) and samples were supplemented with 0.5% Servalyte and 1.2% DeStreak Reagent (GE-Healthcare). Finally, 330 *µ*l of Rehydration buffer (6 M urea; 2 M thiourea; 2% Chaps; 1.2% DeStreak Reagent; 0.5 v/v % Servalyte 3–10 Iso-Dalt for 2D, Roche Complete Protease Inhibitor Cocktail, Pefablock SC Protease Inhibitor) was added to each sample.

### 2.6. 2D-DIGE

For the 2D-DIGE experiment, samples were loaded directly after labeling onto six 24-cm IPG strips pH 3–10NL from Serva (*T* = 4%, *C* = 2.7%) using in-gel rehydration for sample application. Therefore, the IPG strips were rehydrated with 450 *µ*l sample for 16 hours at room temperature. Isoelectric focusing (IEF) was performed for 83 kVh in total. All steps were limited with 75 *µ*A per strip and performed at 20°C (IEF 100 focusing unit, Hoefer Medical Supply).

Protein separation in the second dimension was carried out on precast 2D HPE gels (*T* = 12.5%, *C* = 2.7%) with a SDS-Glycine-Tris buffer system over night. A molecular weight standard, commercially available from Serva, was previously labeled with Cy2. The standard comprising of masses corresponding to 97, 67, 45, 29, 21, 12.5, and 6.5 kDa, respectively, was applied to the gel and positioned next to the IPG strips. The six 2D-DIGE gels were run at two days with 3 gels each (HPE FlatTop Tower from Serva with a Multi-TempIII Thermostatic Circulator and a HPE-Power Supply 1500).

### 2.7. Image Analysis

To visualize the labeled and separated proteins after electrophoresis, the 2D-DIGE gels were scanned at a resolution of 100 *µ*m with a Typhoon FLA 9500 (GE Healthcare). Subsequently, to visualize the total protein load, 2D-DIGE gels were fixed (1% citric acid, 30% ethanol) for 60 min and stained with colloidal Coomassie overnight (5% aluminum sulfate hydrate, 10% ethanol, 0.02% Coomassie G250, and 2% o-phosphoric acid). After destaining with deionized water until appropriate background reduction, gels were scanned with a visual scanner (CanoScan 9900F) using a resolution of 300 dpi.

For image analysis scan files of the 2D-DIGE gels were loaded into DeCyder 2D software (GE Healthcare, version 7.2). Spots were detected with an estimate of 5000 spots for the 2D-DIGE gel. Subsequently, a detection area excluding the region of strip application, molecular weight marker, and running front was determined. Spots with a volume below 50,000 were defined to be background. Stained crumbs originating from the dyes were eliminated by excluding spots with an area below 300. False positive spots, for example, produced by dye artifacts within the gel were removed manually. After editing the gels were normalized towards the Cy2 channel (internal standard).

### 2.8. Statistical Analysis

Spot IDs were allocated to each spot detected and matched in the 2D-DIGE gels. An average ratio of spot volumes from different samples was calculated for each spot ID. An average calculated ratio of 3.0 indicates a 3-fold volume increase when compared to the reference, while an average ratio of −3.0 represents a 3-fold decrease in spot volume. Unchanged spot volumes have ideally an average ratio of 1.0.

The values of 2-fold SD (twice standard deviation) and the corresponding thresholds of spot volumes were calculated and documented. Using filter settings for these threshold values, differences in the spot volumes between two samples were identified. Differences in spot volumes were significant if the average ratio of the compared spot volumes was above the calculated value for 2 standard deviations (2SD; Table 1).

**Figure 2:**
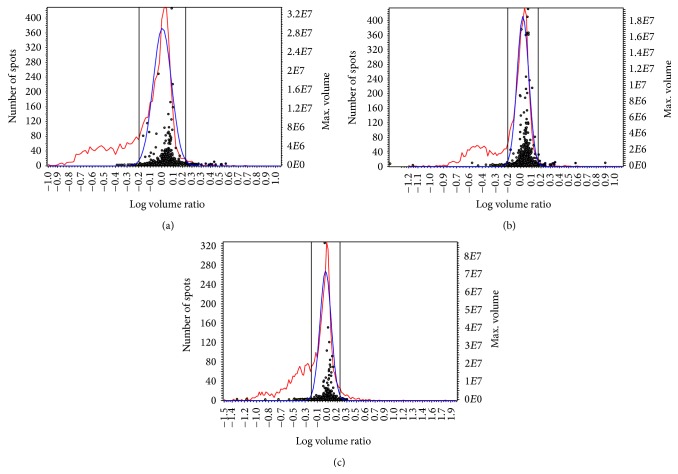
(a) to (c) Comparison of the samples using a 2SD model (SD, standard deviation). Spots upregulated in the first sample of the comparison are depicted in yellow and downregulated spots are depicted in blue. Unregulated spots are shown as grey circles. The threshold spot volume for a regulation (>2SD <−2SD) is shown as grey lines. The red curve represents the frequency distribution of the log volume ratios. The blue curve represents a normalized model frequency fitted to the spot ratios so that the modal peak is zero. (a) Hyperoxia versus control at day 0. Log volume ratio (*x*-axis) ranges from −1.0 to +1.0; number of spots (*y*-axis) ranges from 0 to 420. (b) Hyperoxia versus control at day 3. Log volume ratio (*x*-axis) ranges from −1.2 to +1.0; number of spots (*y*-axis) ranges from 0 to 420. (c) Hyperoxia versus control at day 7. Log volume ratio (*x*-axis) ranges from −1.5 to +1.9; number of spots (*y*-axis) ranges from 0 to 320.

Subsequent data analysis with DeCyder software was performed using a model 2SD in combination with Student's *t*-test to determine differences between the samples. The calculation was based on spot volumes after normalization with the signals from the internal standard sample. Based on the total spot number of detected spots and the standard deviation the DeCyder 2D software calculates the threshold of regulation. In case a spot volume of a certain spot in one sample equates the *x*-fold of the spot volume in a second sample, this spot is regulated if *x* is above the threshold of regulation (*x* > threshold or *x* < threshold). A value for 2SD and a threshold was calculated for each comparison (Figures 2(a)−2(c)).

Using DeCyder 2D software, the normalized spots were matched and the gel images were grouped according to the samples loaded on the gels. Comparison of the samples was performed using the 2SD threshold for regulation and Student's *t*-test. Differences between the samples were defined to be significant if the average ratio of the spot volumes was above or below the threshold for 2SD and the spot did pass Student's *t*-test (*p* = 0.05).

### 2.9. Spot Identification

Specified protein spots were identified with peptide mass fingerprint (PMF) using tryptic in-gel digestion and MALDI-TOF-MS. The spots were manually excised from the specific gels and destained using an acetonitrile buffer. In-gel digestion was performed overnight with 0.05–0.15 *μ*g trypsin (Serva, sequencing grade, category number 37283) in NH_4_HCO_3_ buffer. Peptides were cocrystallized with matrix (10 mg/ml *α*-cyano-hydroxycinnamic acid) onto the MALDI target. Measurement was performed with a 4800 MALDI TOF/TOF Analyzer (AB Sciex) using positive reflector mode (detection range* m/z* 700–4500).

Raw data were processed with Data Explorer software (version 4.9, AB Sciex). All spectra were externally calibrated using a peptide calibration standard. The measured monoisotopic peptide masses were compared to all sequences of SwissProt database, species* Rattus norvegicus*, using the software Mascot (Matrix Science).

For all samples automatically additional MS/MS measurements by fragmenting the five most intense monoisotopic masses from the PMF spectrum were performed. The measured monoisotopic peptide masses and the fragmentation data (MS/MS) were combined for the Mascot search.

Mascot search (peptide mass fingerprint) against rat sequences of SwissProt (http://www.uniprot.org/uniprot/) was performed with the following settings: Enzyme = “trypsin,” fixed modifications = “carbamidomethyl at cysteine,” and variable modifications = “oxidation of methionine.” Monoisotopic mass values with an unrestricted protein mass using a peptide mass tolerance of ±100 ppm were used for identification. For the Mascot database search of the PMF MS spectrum, protein hits with scores greater than 56 were considered significant (*p* < 0.05) (ions score is −10*∗*log(*P*), where *P* is the probability that the observed match is a random event). In case no proteins could be identified from the first spectrum, additional database searches of the automatically generated MS/MS spectra were performed (analog: *p* < 0.05).

### 2.10. Functional Network Mapping and Molecular Pathway Analysis

In order to understand the biological context of the detected regulation of the renal proteome, a systematic functional network mapping and molecular pathway analysis was carried out (Ingenuity Pathways Analysis, IPA, v3.1; Ingenuity Systems; Redwood City, CA, USA). This web-based application enables the exploration of pathway networks relevant to experimental protein expression array data [20]. First, the differentially regulated proteins (focus proteins) were uploaded and used as starting points for building biological networks. Based on relevant protein-protein interactions known from the published data, IPA then determined the interactions between the focus proteins and all other proteins in the IPA-associated database.

After mapping, IPA calculated a score for the network according to the fit of the protein set which quantifies the probability of the focus proteins being found together in a network at random distribution; values above 2.0 reflect a confidence of at least 99% for the network map which is not coincidence. Finally, biological functions assigned to each network were ranked according to the significance of the individual function to the network. *p* < 0.05 was considered statistically significant.

### 2.11. PathwayStudio

PathwayStudio (online version; Elsevier, UK) is a decision support tool, helping researchers analyze and visualize disease mechanisms derived from published scientific data. Organizations use PathwayStudio to support target discovery and validation, to better understand the underlying mechanisms of disease in the context of literature-based and experimental evidence. PathwayStudio offers molecular interactions tagged to viewable sentences, along with biological inference tools that help unravel hidden biology in experimental data or literature-based evidence. It incorporates normalization and statistical tools along with flexible importers to enable analysis and visualization of gene expression, proteomics, and metabolomics data.

## 3. Results

### 3.1. Physiological Parameters

During hyperoxia exposure, inspired oxygen fraction was >0.9 after 3 minutes and >0.98 during the remaining time for the animals in the NH groups while the inspired oxygen fraction of the NN group remained at 0.21 during the whole duration. Besides pO_2_ (581 ± 28 versus 98 ± 12 mmHg; *p* < 0.01), the measured physiological parameters did not differ between groups (NH versus NN).

### 3.2. Protein Expression and Identified Pathways

**Figure 3:**
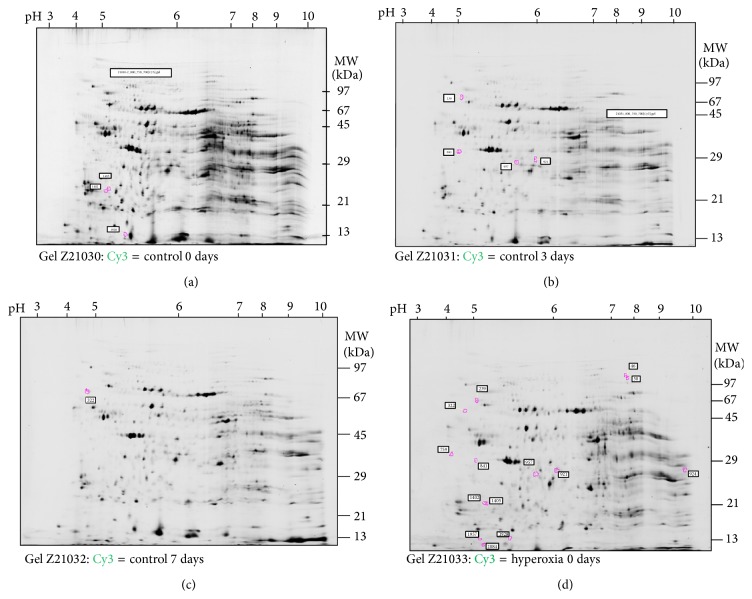
(a) Differences of spot volumes between control and hyperoxia at day 0, found with DeCyder. (b) Differences of spot volumes between control and hyperoxia at day 3, found with DeCyder. (c) Differences of spot volumes between control and hyperoxia at day 7, found with DeCyder. (d) Summary of all differences of spot volumes between control and hyperoxia samples as found with DeCyder.

Using these techniques, a total of 2,094 protein spots were identified in the gels. Expression of 14 proteins was significantly altered: 8 proteins were downregulated (MEP1A_RAT (*p* = 0.012), RSSA_RAT (*p* = 0.035), F16P1_RAT (*p* = 0.011), STML2_RAT (*p* = 0.011), BPNT1_RAT (*p* = 0.011), LGMN_RAT (*p* = 0.011), ATPA_RAT (*p* = 0.03), and VDAC1_RAT (*p* = 0.03)) and 6 proteins were upregulated (MTUS1_RAT (*p* = 0.022), F16P1_RAT (*p* = 0.042), ACTG_RAT (*p* = 0.042), ACTB_RAT (*p* = 0.042), 2ABA_RAT (*p* = 0.012), and RAB1A_RAT (*p* = 0.033); Figures 3(a)–3(d); Table 2). Most proteins were significantly regulated at day 3. In contrast, at day 0 and day 7 only few proteins were regulated. MEP1A and VDAC1 were both significantly downregulated at day 3 (IF, −4.17 and −1.81). Five proteins reached statistical significance but were not able to be identified by the techniques used (Table 2).

**Figure 4:**
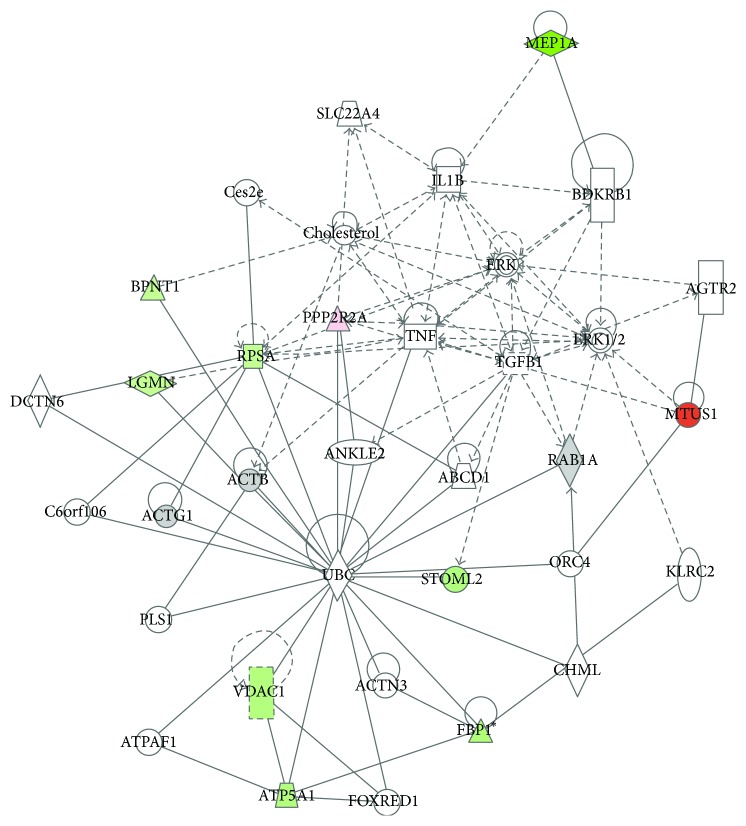
Exemplary protein network identified by IPA at day 3. MEP1A, BPNT1, PPP2R2A, RPSA, LGMN, MTUS1, STOML2, VDAC1, and FBP1 were considered focus proteins being significantly expressed. MEP1A is associated with interleukin. Also, TNF and TGF are members in the network. Green = downregulation; red = upregulation.

Using IPA, several profoundly affected pathways were identified. MEP1A, BPNT1, PPP2R2A, RPSA, LGMN, MTUS1, STOML2, VDAC1, and FBP1 were considered by IPA as focus proteins with a significant influence in the pathways identified (Figure 4).

Using the significantly regulated proteins, IPA identified affected cellular pathways in association with hyperoxia in the kidney: tight junction signaling (*p* < 0.00197) and interleukin signaling (*p* < 0.00318). Additionally, macrophage-stimulating protein/RON receptor signaling pathway (*p* < 0.000567) and remodeling of epithelial adherence junctions pathway (*p* < 0.000961) were identified as significantly involved in the protein alterations found during hyperoxia. In view of diseases and disorders, the regulated proteins were associated with an inflammatory response (*p* < 0.00995), renal disease (*p* < 0.00995), and cell death (*p* < 0.0214) as well as renal necrosis/cell death (*p* < 0.0214).

Additionally, IPA associated the regulated proteins of the present study to the following diseases/functions in the organism (only significantly regulated proteins are listed): organismal death (BPNT1, ACTG1, ACTB, VDAC1, and RPSA), proliferation of cells (ATP5A1, BPNT1, FBP1, MTUS1, RAB1A, RPSA, VDAC1, ACTB, and ACTG1), necrosis (LGMN, MEP1A, PPP2R2A, RPSA, STOML2, VDAC1, ACTB, and ATP5A1), and cell proliferation (RAB1A, RPSA, BPNT1, and RAB1A).

**Figure 5:**
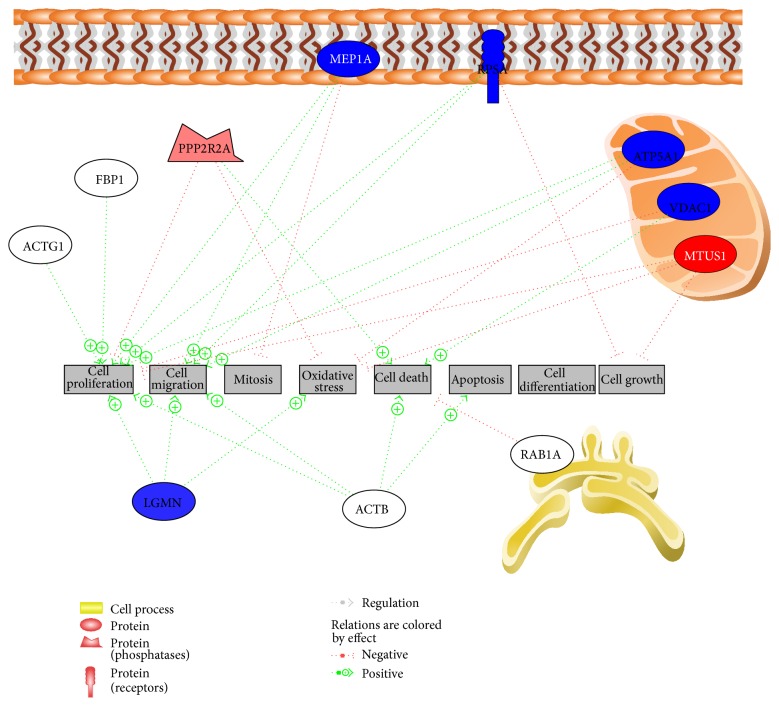
Protein interaction 3 days after hyperoxia as identified by PathwayStudio (Elsevier, UK). Blue = downregulation; red = upregulation.

Using PathwayStudio, a topic map of proteins present at day 3 was generated (Figure 5). Eight different cellular functions were identified: cell proliferation (stimulated), cell migration (stimulated), mitosis (inhibited), oxidative stress (inhibited), cell death (stimulated), apoptosis (stimulated), cell differentiation, and cell growth (inhibited). All cellular functions could be associated with oxidative stress.

Concerning toxicological functions, renal necrosis and cell death were found to be significantly associated with the protein alterations observed (*p* < 0.00214).

## 4. Discussion

In order to analyze the effect of hyperoxia treatment on rat renal tissue, rat kidneys from animals at 0, 3, or 7 days after hyperoxia or respective control treatment were used. Subsequent analysis discovered 14 low abundant protein spots (Table 2) that differed significantly in the hyperoxia and control samples. Interestingly, more differences were found at 3 days after hyperoxia treatment (4 spots) compared to those at 7 days after treatment (1 spot) or at day 0 (3 spots). Meprin A (MEP1A) and voltage-dependent anion-selective channel protein 1 (VDAC1) seem to be the most interesting renal tissue molecules after hyperoxic exposure. Both proteins were downregulated at day 3 with VDAC1 remaining downregulated at day 7.

### 4.1. General Hyperoxia Considerations

Prior studies have demonstrated various short- and long-lasting effects of short-term as well as long-term hyperoxia on several organs [4, 5, 7, 8], including the kidney [6]. However, the results of several recent studies are inconclusive [13, 14, 21].

Popescu et al. have previously shown in a rat model that a preterm hyperoxic insult results in a reduced nephron number in adulthood. In another study of that group, hyperoxia exposure resulted in a significant reduction in both nephrogenic zone width and glomerular diameter and a significantly increased apoptotic cell count [6]. Overall, this study supports the premise that prematurely born neonates exposed to high oxygen levels after birth are vulnerable to impaired renal development [6]. Moreover, normobaric hyperoxia created mild increases in renal tubular necrosis, dilation, regeneration, and interstitial inflammation [7].

On the other hand, Sutherland and colleagues [14] analyzed the effect of long-acting hyperoxia in neonatal mice but found no overt long-term effects of early life hyperoxia on glomerular structure. Also Rostami et al. found no significant impact of hyperoxia on protection from delayed graft function [13]. Moreover, other authors even postulated a protective effect on the kidneys through hyperoxic preconditioning [21].

### 4.2. Meprin A

Meprin was first discovered in the early 1980s. Meprins are zinc-dependent metalloproteinases of the astacin family that were initially isolated and characterized from brush-border membranes of the rat kidneys [22–24]. They are abundantly expressed at the apical membranes of the proximal tubules in the corticomedullary junction and comprise 5% of the total proteins of kidney brush-border membranes [23, 25].

The two isoforms Meprins A and B are highly regulated and secreted. They are cell-surface homo- and heterooligomeric enzymes [26]. Recent progress in identifying the ability of Meprin to degrade extracellular matrix (ECM) proteins, to process proinflammatory cytokines, and to promote leukocyte infiltration has revived further interest in studying Meprin in a wide range of diseases, from acute kidney injury (AKI) to inflammation [23]. There is little information regarding the role of Meprin A in the pathogenesis of significant renal syndromes such as acute renal failure (ARF). Besides the fact that high concentrations of Meprin may be considered as a biological marker of ARF, little is known in conditions with reduced expression of Meprin, as observed in the present study. MEP1A was downregulated only at day 3 (IF −4.17). Additionally, IPA revealed an association of MEP1A with tubular necrosis and the protein was linked to interleukin expression (Figure 4). However, Meprin A redistribution has been attributed to cellular damage and promotion of inflammatory response [27].

Other studies also reported that a reduced expression of Meprin is detectable in inflammatory processes, for example, in the bowel during colitis [28]. Keiffer and Bond demonstrated that Meprins A and B cleave IL-6 to a smaller product and, subsequently, are capable of extensive degradation of the cytokine [29]. These results are consistent with the proposition that one function of Meprin metalloproteases is to modulate inflammation by inactivating IL-6 [29].

Therefore, reduced expression of Meprin in the present study could at least give an indication that there is a link between hyperoxia and a cellular response in the kidney.

### 4.3. VDAC1

VDAC1 is an outer mitochondrial membrane (OMM) protein, is crucial for regulating mitochondrial metabolic and energetic functions, and acts as a convergence point for various cell survival and death signals [30]. VDAC1 is also a key player in apoptosis, involved in cytochrome c release and interactions with antiapoptotic proteins [30]. Weisthal and colleagues demonstrated that different apoptosis inducers caused VDAC1 overexpression. In contrast, decreasing [Ca^2+^] inhibited VDAC1 overexpression [30].

However, in the present study, VDAC1 was less expressed at days 3 and 7 after hyperoxia as compared to day 0. This could give an indication for altered mitochondrial metabolic and energetic functions after hyperoxia. Additionally, this protein was linked to organismal death, proliferation of cells, and necrosis by IPA in our study.

Besides our own hypotheses, other studies also analyzed the relevance of VDAC. Li and colleagues [31] found VDAC1 to play a significant role in neuroprotection and to attenuate both apoptotic and necrotic molecular pathways. Another group of Chu and colleagues linked VDAC1 to cellular injury in Parkinson's disease [32]. Chen and colleagues found that inhibition of VDAC1 prevents Ca^2+^-mediated oxidative stress and apoptosis in the brain [33]. For the lung, Chacko et al. found that expression of VDAC1 is required for full processing and activation of caspase-8 and supports a role for mitochondria in regulating apoptosis signaling via the death receptor pathway [34].

Knowing these effects of VDAC1, it may be possible that reduced expression of VDAC1 in our study could be an indicator of a cellular countermeasure to hyperoxia due to a higher risk for apoptosis and cellular injury.

### 4.4. Bioinformatic Analyses

Interestingly, more differences were found at 3 days after hyperoxia exposure (4 spots) than at 7 days after exposure (1 spot) or at day 0 (3 spots). However, it is not clear from the present point of view if the hyperoxic protein regulation alterations are limited to the first few days after exposure or if the proteomic technique used in this study failed to identify further regulated proteins at day 7.

Rostami and colleagues [13] also published some evidence for an only short-lasting protein effect of hyperoxia exposure. These colleagues analyzed protein alteration in urine and found most changes after 2-3 days. In their study, neutrophil gelatinase associated lipocalin (NGAL), interleukin (IL1B), tumor necrosis factor (TNF-*α*), and transforming growth factor-ß (TGF-ß) were found to be reliable biomarkers for early diagnosis of kidney injury [13]. Although all these proteins were not found in the kidney tissue of our study, both Ingenuity Pathway Analysis and PathwayStudio found an association with at least IL1B, TNF, and TGF (Figure 4).

In agreement with other studies, our data also suggests that MEP1A, which is connected in the IPA network with IL1B, TNF, and TGF (Figure 4), might be a promising biomarker to detect hyperoxia-induced effects in the kidney. While significantly altered pathways and signaling cascades were identified with the present study approach, it remains unclear if the effects observed are ultimately beneficial or deleterious for renal tissue function.

Therefore, further studies on humans and in other easily available tissues/materials (e.g., urine) are warranted.

### 4.5. Hyperoxia-Induced Protein Alterations in Other Organs

Interactions of superoxide dismutases (SODs) with reactive oxygen species (ROS) have important roles during hyperoxia [35–37]. ROS toxicity has also been shown to be a common feature underlying some respiratory diseases by initiating inflammatory response, destruction of the alveolar-capillary barrier, and impaired gas exchange [38, 39].

In the lung, hyperoxia causes a massive production of reactive oxygen species (ROS), which in turn initiate inflammatory response, destruction of the alveolar-capillary barrier, and impaired gas exchange [38, 39]. The excessive production of ROS under hyperoxic conditions leads to modifications of macromolecules and pulmonary cell death [40, 41]. In the lung, an increased expression of the protein IFIT3 could represent a mechanism of oxygen toxicity induced cell growth inhibition [5, 40, 41]. Furthermore, IFIT3 leads to an inhibition of interferon-*β*-induction and inhibits Nuclear Factor kappa-B (NF*κ*B) dependent gene induction [42]. NF*κ*B serves as a central mediator for detection of stress and marshalling cellular defense systems [5]. These protein alterations may counteract immune signaling of the lung and were recently associated with cell growth inhibition, regulation of apoptosis, and approval of structural cell integrity [5].

Besides pulmonary complications, cerebral effects during hyperoxia remain to be a focus of prominent interest today, as oxidative metabolism and the generation of ROS play a major role in the central nervous system (CNS) [4, 43]. In the brain, hyperoxia causes a transient but significant decrease in cerebral blood flow (CBF) [44], followed by a later rise [45], and its major biological effects include an induction of molecular stress responses, inflammation, and modulation of cell death and cell growth [4, 46]. Also in the brain, NF*κ*B serves as a central mediator of stress and is inhibited by hyperoxia [4] which also was demonstrated to be associated with inflammation, Alzheimer's disease, oxidative stress, apoptosis, and cell death as well as cell growth, survival, and differentiation pathways [4].

### 4.6. Limitations

Using the molecular techniques presented has certain limitations. Since only low abundant protein spots were found to have changed after exposure to hyperoxia, interpretation of data has to be done carefully. It is always possible that signals may originate from artificial background staining and this can be easily missed if the signal is weak. Since the protein samples are very complex and the high and medium abundant proteins did not seem to be affected by the treatment, a reduction of the complexity could be considered.

Separation of proteins in 2D gels reflecting only a smaller window of the proteome, by, for example, using narrower pH gradients, could reduce sample complexity. However, using a smaller window may veil other proteins at the border of possible detection (e.g., high or low pH or high or low molecular mass).

An increase of the overall protein load could also be considered in order to confirm the changes of low abundant proteins that would then appear above the detection limit.

After analysis and identification of proteins by MALDI-TOF mass spectrometry an additional validation of the altered proteins by Western blot or immunohistochemistry is often used to increase certainty of the results. We were not able to perform such additional validation tests and, therefore, careful interpretation of the data is required. However, by using MALDI-TOF, tandem mass spectrometry, and adjusted statistics (e.g., mascot score, etc.) a high validity is achieved otherwise, used in the present study.

## 5. Conclusions

The detailed characterization of organ-specific responses to hyperoxia is critical to improving our understanding and interpretation of alterations in cellular protein expression of the kidney. In the present study, significant alterations in renal protein expression could be demonstrated up to 7 days even after short-term hyperoxia. Hyperoxia treatment apparently affected only low abundant proteins which were mainly associated with inflammation signaling cascades.

This work has important implications in the use of MEP1A and VDAC1 as potential biomarker candidates to identify hyperoxic injury in kidney cells, for example, after hyperoxia exposure or hyperoxia-induced organ inflammation. Whether Meprin A mediates cellular repair or indicates tubular cell death in the kidney remains to be elucidated.

The altered proteins were identified in the renal tissue. Therefore, future efforts could focus on detecting these proteins in urine to facilitate identification of hyperoxic kidney injuries, for example, in humans, and useful biomarkers for identification of hyperoxia-induced cellular injury.

## Figures and Tables

**Table 1:** Standard deviation for 2D-DIGE gels (2-dimensional difference gel electrophoresis).

Comparison	2SD	Threshold for regulation (2SD)
Hyperoxia 0 day vs. control 0 day	2.11	1.70
Hyperoxia 3 day vs. control 3 day	2.82	1.54
Hyperoxia 7 day vs. control 7 day	2.70	1.78

SD = standard deviation; vs. = versus.

**Table 2:** Proteins significantly regulated and identified by MALDI-TOF with characteristic features and induction factors (IF) at day 0 (T0), day 3 (T3), and day 7 (T7).

Spot number	Figure	Molecular weight [kDa]	Significance, *t*-test	Protein Mascot score	Protein name	Accession number	T0	T3	T7
46	Figure 3(d)	~100	0.0026		Not identified	—	n.s.	n.s.	n.s.

50	Figure 3(d)	~100	0.036		Not identified	—	n.s.	n.s.	n.s.

239	Figure 3(b)	72	0.012	71	Meprin A subunit alpha	MEP1A_RAT	n.s.	−4.17	n.s.

322	Figure 3(c)	70	0.022	23	Microtubule-associated tumor suppressor 1 homolog	MTUS1_RAT	n.s.	8.12	8.12

759	Figure 3(d)	33	0.035	90	40S ribosomal protein SA	RSSA_RAT	−1.77	−1.77	−1.77

841	Figure 3(b)	30	0.012		Not identified	—	n.s.	n.s.	n.s.

921	Figure 3(b)	29	0.011	202	Fructose-1,6-bisphosphatase 1	F16P1_RAT	n.s.	−1.87	n.s.
					Stomatin-like protein 2, mitochondrial	STML2_RAT			
					3′(2′),5′-bisphosphate nucleotidase 1	BPNT1_RAT			
					Legumain	LGMN_RAT			

924	Figure 3(d)	28	0.03	64	ATP synthase subunit alpha mitochondrial	ATPA_RAT	n.s.	−1.81	−1.81
					Voltage-dependent anion selective channel protein 1	VDAC1_RAT			

957	Figure 3(b)	28	0.042	256	Fructose-1,6-bisphosphatase 1	F16P1_RAT	n.s.	n.s.	1.74
					Actin, cytoplasmic 2	ACTG_RAT			
					Actin, cytoplasmic 1	ACTB_RAT			

1402	Figure 3(a)	23	0.018	60	Serine/threonine-protein phosphatase 2A 55 kDa regulatory subunit B alpha isoform	2ABA_RAT	1.74	1.74	1.74

1405	Figure 3(a)	23	0.012	60	Serine/threonine-protein phosphatase 2A 55 kDa regulatory subunit B alpha isoform	2ABA_RAT	1.74	1.74	1.74

1928	Figure 3(a)	14	0.033	59	Ras-related protein Rab-1A	RAB1A_RAT	1.81	n.s.	n.s.

1957	Figure 3(d)	14	0.0028		Not identified	—	n.s.	n.s.	n.s.

2048	Figure 3(d)	13	0.0096		Not identified	—	n.s.	n.s.	n.s.
